# A possible space-based tsunami early warning system using observations of the tsunami ionospheric hole

**DOI:** 10.1038/srep37989

**Published:** 2016-12-01

**Authors:** Masashi Kamogawa, Yoshiaki Orihara, Chiaki Tsurudome, Yuto Tomida, Tatsuya Kanaya, Daiki Ikeda, Aditya Riadi Gusman, Yoshihiro Kakinami, Jann-Yenq Liu, Atsushi Toyoda

**Affiliations:** 1 Department of Physics, Tokyo Gakugei University, Tokyo, Japan; 2 Earthquake Research Institute, the University of Tokyo, Japan; 3 Department of Engineering for Innovation, National Institute of Technology, Tomakomai College, Hokkaido, Japan; 4 Graduate Institute of Space Science, National Central University, Chung-li, Taiwan; 5 Nuclear Safety Research and Development Center, Chubu Electric Power Co., Omaezaki, Japan

## Abstract

Ionospheric plasma disturbances after a large tsunami can be detected by measurement of the total electron content (TEC) between a Global Positioning System (GPS) satellite and its ground-based receivers. TEC depression lasting for a few minutes to tens of minutes termed as tsunami ionospheric hole (TIH) is formed above the tsunami source area. Here we describe the quantitative relationship between initial tsunami height and the TEC depression rate caused by a TIH from seven tsunamigenic earthquakes in Japan and Chile. We found that the percentage of TEC depression and initial tsunami height are correlated and the largest TEC depressions appear 10 to 20 minutes after the main shocks. Our findings imply that Ionospheric TEC measurement using the existing ground receiver networks could be used in an early warning system for near-field tsunamis that take more than 20 minutes to arrive in coastal areas.

Approximately 230 thousand and 20 thousand people died as a result of the 2004 M9.2 Sumatra–Andaman Earthquake (Sumatra EQ) and the 2011 M9.0 off the Pacific Coast of the Tohoku Earthquake (Tohoku EQ). Most of the victims were killed by the earthquake-induced tsunamis. This indicates that the present forecasting systems of tsunami height are not effective, even in Japan, which is equipped with a dense scientific observation network. To estimate the initial tsunami height of an EQ with a magnitude greater than 8 from seismic data, records of long-period seismic waves for, say, approximately 10 minutes are required to compute the vertical displacement of the rupture area. Because additional tens of minutes are required for accurate computation of the rupture area, it is difficult to issue a warning of the precise height of the forthcoming near-field tsunami sufficiently early for residents in coastal areas to evacuate. To overcome this problem, offshore monitoring for tsunamis using global positioning system (GPS) buoys and ocean-bottom pressure gauges has been deployed^1^. However, a sufficiently dense network for such a monitoring system would require a high budget and human power^2^. Thus, less expensive and practical tsunami early warning systems are desired urgently.

Low-frequency acoustic waves, termed infrasonic waves, are excited by sudden displacement of the ground and sea surface resulting from a large EQ and tsunami. When the acoustic waves reach the ionospheric F region, approximately 10 minutes after the main shock, they disturb the ionospheric plasma. This plasma variation was first detected by measurements of ionosondes (radars for the electron density profile in the bottom-side ionosphere)^3^ and high-frequency Doppler sounders (bistatic radars for ionospheric F region variation)^4^ at the 1964 M9.2 Alaska EQ. After developing a method to measure the ionospheric total electron content (TEC) between a GPS satellite and its receivers on the ground, using the phase difference between two carrier signals from the satellite, the ionospheric disturbance can be detected as a TEC disturbance. In particular, after increase in the number of ground-based GPS receivers, spatio-temporal images of the TEC disturbance generated by the EQ and tsunami allow identification of the epicentre^5^ and tsunami propagation^6^,^7^,^8^,^9^. The TEC disturbance generated above the epicentre radially propagates with the velocity of acoustic waves in the ionosphere, e.g. approximately 1 km/s^9^,^10^. The intensity of southward (northward) propagation of the TEC disturbance was clearly visible in the northern (southern) hemisphere, while that of northward (southward) propagation was not clearly observed, because the asymmetrical intensity of northward and southward propagation is caused by the different plasma motion forced to the direction of magnetic field lines^11^.

For the Tohoku EQ, the TEC disturbance began above the tsunami source area and the TEC disturbance originating from acoustic waves subsequently propagated radially. Subsequently, the acoustic and gravity waves generated by Rayleigh waves and tsunami propagations caused the TEC disturbances^9^,^10^,^12^,^13^. In addition to the origin of these disturbances, a TEC depression with a radius of a few hundred kilometres, which remained stationary for approximately 1 hour, was observed^14^. This was termed as the tsunami ionospheric hole (TIH). A TIH was also visible after the Sumatra EQ and the 2010 M8.8 Chile EQ. Further investigations revealed that TIHs appeared after other megathrust and shallow global EQs with magnitudes greater than 7.2^15^. A numerical simulation of the TIH^16^ elucidated the plasma dynamics. When the acoustic waves reached the ionosphere, the amplitude of the acoustic waves was intensified due to the very low density of neutral atmosphere. The acoustic waves forced the ionospheric plasma to considerably migrate only along the magnetic field line because of neutral-ion drag. Subsequently, in the decompression phase of the acoustic waves, the downward plasma caused dissociative recombination and suppressed ion production, creating a TEC depression, i.e. TIH. The immediate recombination and slow production caused the TIH to exist for a long time.

Monitoring of tsunami-generated TEC disturbances using GPS receiving network has been expected to be a practical tsunami early warning system, because the disturbance can be detected about 10 minutes after the main shock and the GPS receiving network is improving^17^,^18^ Moreover, the quantitative relationship between initial tsunami height and TEC disturbance amplitude expected from the physical point of view might provide a numerical warning. In fact, Astafyeva *et al*.^15^ reported a correlation not only between initial tsunami height and initial TEC enhancement amplitude, but also between initial tsunami height and TIH duration for 11 large global EQs.

Thus, the quantitative relationship between the initial tsunami height and amplitude of the TEC disturbance immediately above the tsunami source area is required to assess the possibility of using GPS-TEC ionospheric monitoring for a practical early warning system. In this paper, we focus on the value of a TEC depression in a TIH to estimate initial tsunami height, because the TIH remains stationary. We discuss the possibility of constructing an early warning system using only the existing GPS network.

## Results

**Figure 1:**
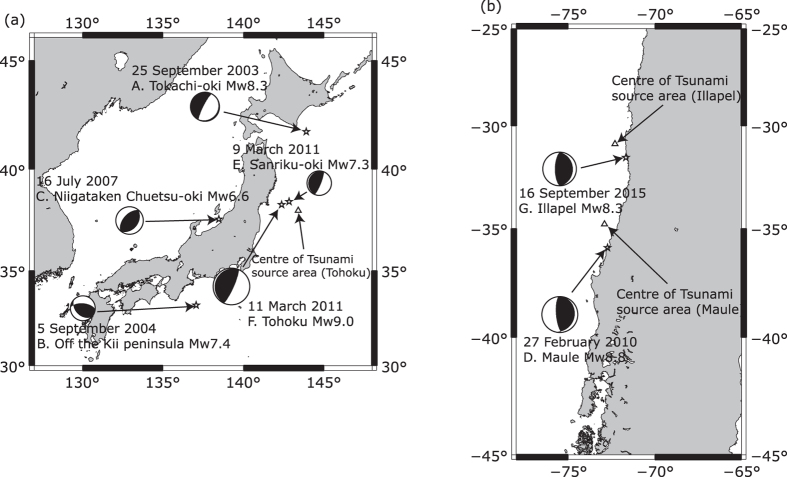
Locations of EQs and their focal mechanisms. (**a**) Japan. The grey line denotes the tsunami source area of the Tohoku EQ. (**b**) Chile. The map was drawn using GMT 5 (http://gmt.soest.hawaii.edu/).

In the analysis herein, we investigate large EQs with accompanying tsunamis in Japan and Chile because the dense and intermediate-dense networks of GPS receiving stations enables us to obtain TEC data above the tsunami source area in magnetic latitudes ranging from 20°N to 35°N in Japan and from 20°S to 35°S in Chile, and the inclination of the magnetic field can be regarded as approximately constant. After commencement of the GPS-TEC observation, seven EQs with sub-ionospheric points (SIPs; the footprint of intersection between the ionosphere modelled as a thin layer and the path of a GPS satellite and a receiver, see information on GPS data in the Method section) around the tsunami source area occurred in Japan and Chile: the 2003 M8.0 Tokachi-oki EQ and the 2004 M7.4 off the Kii peninsula EQ, the 2007 M6.6 Niigataken Chuetsu-oki EQ, the 2010 M8.8 Off Shore Maule EQ (the Maule EQ), the 2011 M7.0 Sanriku EQ (the largest foreshock of the Tohoku EQ), the 2011 M9.0 Tohoku EQ, the 2015 M8.3 Illapel EQ, (Fig. 1 and Table 1). The initial tsunami heights of these EQs were seismologically estimated^19^,^20^,^21^,^22^,^23^,^24^,^25^,^26^,^27^,^28^,^29^,^30^,^31^,^32^.

**Figure 2:**
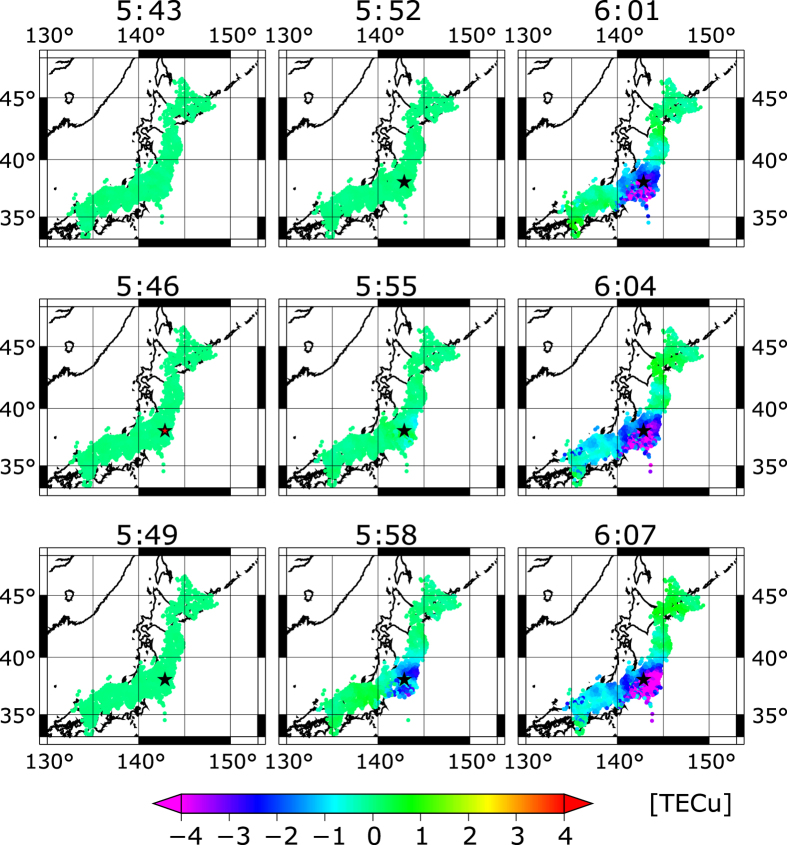
Snapshot images of spatial distribution of ΔvTEC every 3 minutes before and after the main shock of the Tohoku EQ. The star indicates the epicentre. The map was drawn using GMT 5 (http://gmt.soest.hawaii.edu/).

To discriminate the TEC trend (mainly originating from the variable length of the slant path between the satellite and the receiver) and the tsunami-generated TEC disturbance including the TIH and the acoustic waves, the difference between the TEC trend and the raw TEC data was analysed. Considering the elevation angle between the ground and the slant path, the residual component (termed ΔvTEC) at the SIP was obtained. After removing the acoustic wave component using a low-pass filter (LPF), the TIH component of ΔvTEC, termed LPF ΔvTEC, was obtained. As an example, spatial images of LPF ΔvTEC before and after the main shock of the Tohoku EQ are illustrated in Fig. 2.

**Figure 3:**
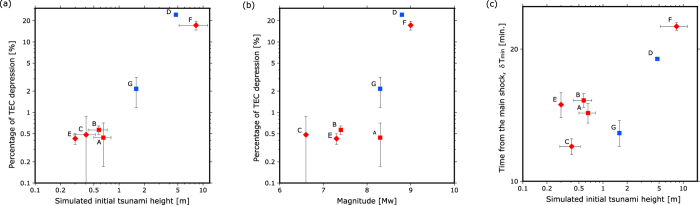
Relationship between percentage TEC depression and initial tsunami height and magnitude. A, B, C, D, E, F and G denote the 2003 M8.0 Tokachi-oki EQ and the 2004, M7.4 Off the Kii peninsula EQ, the 2007 M6.6 Niigataken Chuetsu-oki EQ, the 2010 M8.8 Maule EQ, the 2011 M7.0 Sanriku EQ, the 2011 M9.0 Tohoku EQ, and the 2015 M8.3 Illapel EQ. Red and blue means EQ in Japan and Chile, respectively. Rhombus and squares mean daytime (6–18 LT) and nighttime EQs in local time. (**a**) Initial tsunami height versus percentage TEC depression. (**b**) Magnitude versus percentage TEC depression. (**c**) Initial tsunami height versus time after main shock (ΔT_min_).

We focused on the percentage TEC depression caused by the TIH and the interval δT_min_ between the time of the main shock and the minimum value of LPF ΔvTEC (namely the largest TIH). The relationship between the percentage of the TEC depression, δT_min_, initial tsunami height and EQ magnitude are illustrated in Fig. 3. As shown in Figs 3a and b, the percentage of TEC depression versus the initial tsunami height are correlated. In contrast, the percentage of TEC depression and the magnitude are not correlated, which is convincing because, in general, the magnitude of the tsunami-generating EQ is not always proportional to the initial tsunami height^33^.

In Fig. 3a, the Maule EQ (D) is slightly large, probably because the night-time small background TEC yielded relatively large error of the percentage of TEC depression, while δT_min_ of the Maule EQ shown in Fig. 3c seem appropriate. On other hand, in Fig. 3c, the four small EQs with small simulated initial tsunami height (A, B, C, and E) seems inappropriate, probably because of difficult identification for the small depression, while the percentage of TEC depression for them are relatively appropriate. Thus, the EQ with more than 1 m initial tsunami height during daytime might show precise percentage of TEC depression and δT_min_.

## Discussion

Estimation of the initial tsunami height after large EQs such as the Tohoku EQ takes approximately 20 minutes only from the correlations shown in Fig. 3a and c. This means that an early warning system using TEC measurements may be useful for some coastal areas if 20 minutes plus the necessary time for evacuation is longer than the time taken for the near-field tsunami to arrive at the coast.

The correlations shown in Fig. 3a and c will be useful to verify the simulation of Shinagawa *et al*.^16^. After parameterization of the simulation, it is possible to predict the initial tsunami height in other regions with different magnetic inclination. As a spatial ΔvTEC map gradually shifts with satellite movement and the number of visible GPS satellites is limited, the ΔvTEC map does not provide continuous coverage of an entire area even in Japan. As the number of other satellites and receivers of Global Navigation Satellite Systems (GNSS) such as GLONASS, BeiDou, and Galileo and Regional Navigational Satellite System (RNSS) such as QZSS are rapidly increasing, TEC measurements of GPS and other GNSS/RNSS are expected to continuously cover the entire area in the near future. In addition, real-time GPS data is currently provided in many countries. In the present methodology, ΔvTEC can be immediately calculated even using personal computer after obtaining real-time slant TEC. This yields a feasible early warning system for the near-field tsunami.

## Method

### GPS data

GPS is a satellite navigation system that provides precise location and time information derived from the phase differences of carrier radio signals (1575.42 and 1222.60 MHz) between several satellites and their receivers. Ionospheric plasma, the dispersive medium for radio signals, causes a frequency-dependent phase delay during signal propagation. The phase difference between two carrier signals measured at the receivers is proportional to the TEC between the satellite and the receiver, termed the slant TEC. The unit of TEC value is TECu, corresponding to 1.0 × 10^16^ electron m^−2^. The altitude of the F region, which is the major contributor to TEC, is approximately 200 to 400 kilometres. For the sake of simplicity, we approximated the region as a thin layer at 300 kilometres. The point at which the slant path from the satellite to the receiver intercepts this ionospheric layer is termed the ionospheric point. In Japan, GPS data are provided by the Geographical Survey Institute of Japan, which has installed more than 1200 receivers in a nationwide GPS array, named the GPS Earth Observation Network (GEONET; ftp://terras.gsi.go.jp/). Its sampling period of GPS data is 30 seconds or 1 second. GEONET provides spatial-temporal ionospheric images. In Chile and Argentina, GPS data are provided by UNAVCO (ftp://data-out.unavco.org/) and the Instituto Geográfico Nacional (http://www.ign.gob.ar/NuestrasActividades/Geodesia/Ramsac/DescargaRinex), which has installed several tens of receivers in a GPS array, having sampling periods of GPS data between 15 and 60 seconds.

### Percentage of TEC depression in TIH

To derive the TEC disturbance caused by the TIH, the following procedure was applied. First, the least-squares quadratic curve of the slant TEC time-series in the period from 30 minutes prior to 40 minutes after the main shock was obtained, except for the Tohoku EQ and the Maule EQ (Fig. S1a). For the Tohoku and Maule EQs, only the period from 30 minutes prior to 7 minutes after the main shock was used because the TIH was too large to obtain appropriate approximation after the TIH. Peculiar bias components for each satellite and the receivers were reduced by the difference between this fitting curve and the raw data. Second, the difference in the vertical TEC (i.e. ΔvTEC) was derived by multiplying the cosine between the slant direction and the satellite earth direction by the difference between the fitting curve and the raw data (Fig. S1b).

A spectrum analysis of ΔvTEC in the Tohoku EQ for all stations is illustrated in Fig. S2. The period of the spectrum analysis was 4096 seconds, consisting of 1024-second data of ΔvTEC located in a constant part of a boxcar window function. In Fig. S2, the vertical axis indicates the distance between the SIP at the time of the main shock and the centre of the tsunami source area, and the horizontal axis indicates the period of the spectrum. Several resonant modes of acoustic waves occurred; in particular, they were intense in the period from 150 to 210 seconds above the tsunami source area. According to Watada and Kanamori^34^, the observed distinct patterns from 150 to 210 seconds are inferred to be resonant modes of the acoustic waves excited by the tsunami. To exclude the variations of resonance modes including the initial TEC pulse, i.e. the acoustic wave components, and extract the component of the TIH, the ±150 sec running mean of ΔvTEC except the Niigataken Chuetsu-oki EQ was taken, i.e. LPF ΔvTEC (Fig. S2b). For the Niigataken Chuetsu-oki EQ, ± 90 sec running mean is used because of a small TEC disturbance.

The following procedure was employed to estimate the value of the TEC depression of the TIH above the tsunami source area. First, the tsunami source area was defined to be the average location of the Tohoku EQ (N38.0, E143.4), the Maule EQ (S34.8, W72.9), and the Illapel EQ (S30.9, W72.3) reported by several articles^28^,^29^,^30^,^31^,^26^,^27^,^32^, and equivalent to the epicentre of other small EQs. Time-series of LPF ΔTEC to estimate the TEC depression were selected such that the SIP at the time 7 min after the main shock was located within 100 kilometres from the tsunami source area of Mw ≥ 8 EQs and 50 kilometres from the epicentre of the other EQs from 7 minutes to 24 (the Tohoku and Maule EQs) and 16 (the other EQs) minutes after the main shock (Fig. S1). We defined δvTEC^TIH^ as the difference between the value of the LPF ΔvTEC 7 minutes after the main shock and the minimum value of LPF ΔvTEC, and δT_min_ as the interval between the main shock and the minimum time of LPF ΔvTEC. Considering the simulation of Shinagawa *et al*.^16^, the TEC depression of TIH was assumed to be proportional to the electron density in the F2 region immediately before the TEC disturbance, because the depressed plasma migrates downward from the F2 region. In this study, the percentage TEC depression with respect to absolute vTEC, i.e. δvTEC^TIH^/vTEC × 100, was discussed for comparison with different solar conditions such as during different seasons and at different local times. For derivation of absolute vTEC, data of Global Ionosphere Maps Total Electron Content (GIMTEC: ftp://ftp.unibe.ch/aiub/CODE/) was used. Finally, the average values and standard deviations of the percentage TEC depression (δvTEC^TIH^/vTEC × 100) and δT_min_ for each EQ from the selected time-series of LPF ΔvTEC (Figs S1 and S2) were obtained.

### Geomagnetic storm

Ionospheric storm caused by geomagnetic storm causes TEC variation. If the dominant quarter period of TEC variation during ionospheric storm is much longer than 15 min corresponding to the interval between the acoustic wave arrival at the ionosphere and the minimum of TEC caused by the TIH, the percentage TEC depression is not significantly affected by the ionospheric storm because the background TEC values during the interval are regarded as the same. In fact, the dominant period of TEC variation during ionospheric storm is on the order of hours^35^. In contrary, the TEC variations with the period of less than 300 s are reduced by the running mean process. However, the periods for more than 300 s should be taken into account. Only in the Tohoku EQ for the present analysis, Dst index was large (Table 1). Spectrum analysis shown in Fig. S2 indicated no significant variation for period larger than 300 s.

### Earthquakes with tsunami

The earthquakes accompanying tsunami listed in Table 1 are selected as follows: 1) Geomagnetically mid-latitude are selected because the numerical simulation^16^ was applied for these area. 2) Accessible GPS data has been provided. 3) At least one SIP 7 min after the mainshock existed within 50 km and 100 km epicentral distances for M < 8 and M ≥ 8. 4) The initial tsunami height (> 0.1 m) was reported in the refereed journal except the largest foreshocks of the Tohoku EQ, which is reported below.

### Initial tsunami height

Average values and standard deviations of the initial tsunami height were obtained from sources listed in references^19^,^20^,^21^,^22^,^23^,^24^,^25^,^26^,^27^,^28^,^29^,^30^,^31^,^32^. Most studies were consistent with our results, comparing tsunami heights observed at several coastal tide gauges and offshore GPS buoys with the simulated values and defining the initial tsunami height as the vertical deformation from fault motion.

Tsunami waveforms recorded at tide gauges and offshore tsunami gauges can be used in tsunami waveform inversion to estimate the initial tsunami height in the source area. For the case of the largest foreshock of the 2011 Tohoku EQ that occurred on 9 March, five tsunami waveforms at two offshore pressure gauges and three GPS buoys were used to estimate the slip distribution of the largest foreshock^36^. This slip distribution has a major slip region (measuring 45 km × 45 km) with slip amounts larger than 0.5 m. The seismic moment calculated from the slip distribution is 1.2 × 10^20^ Nm (Mw 7.3), which is consistent with the United States Geological Survey Wphase solution. The maximum initial tsunami height calculated from the slip distribution is approximately 0.3 m (Fig. S5).

## Additional Information

**How to cite this article**: Kamogawa, M. *et al*. A possible space-based tsunami early warning system using observations of the tsunami ionospheric hole. *Sci. Rep.* **6**, 37989; doi: 10.1038/srep37989 (2016).

**Publisher's note:** Springer Nature remains neutral with regard to jurisdictional claims in published maps and institutional affiliations.

## Supplementary Material

Supplementary Information

## Figures and Tables

**Table 1:** List of EQs, initial tsunami heights, and Dst index (http://wdc.kugi.kyoto-u.ac.jp/).

	Earthquake	Date	Time (UT)	Magnitude (Mw)	Depth (km)	Epicentre	Initial tsunami height (m)	Dst index
A	Tokachi-okiEQ	25 Sep. 2003	19:50:06	8.3	27	41.775°N	0.8 m 19	−34
						143.904°E	0.5 m 20	
B	Off the Kii peninsula EQ	5 Sep. 2004	14:57:18	7.4	10	33.216°N	0.5 m^21^	−5
						137.061°E	0.6 m 22	
							0.4 m 23	
							0.8 m 24	
C	Niigataken Chuetsu-oki EQ	16 July 2007	1:16:23	6.6	10	37.576°N	0.51 m 25	−21
						138.469°E	0.53 m 25	
							0.29 m 25	
							0.29 m 25	
D	Maule EQ	27 Feb. 2010	06:34:14	8.8	35	35.909°S	4.5 m 26	−3
						72.733°W	5 m 27	
E	Sanriku-oki EQ(Largest foreshock of the Tohoku EQ)	9 March 2011	02:45:20	7.3	32	38.440°N	0.3 m	−17
						142.840°E	(Fig. S5)	
F	Tohoku EQ	11 March 2011	05:46:24	9.0	30	38.297°N	8 m 28	−83
						142.372°E	7 m 28	
							6 m 28	
							7 m 29	
							15 m 30	
							6.5 m 31	
G	Illapel EQ	16 Sep. 2015	22:54:32	8.3	22.4	31.573°S	1.6 m 32	−19
						71.674W		
