# Supplementary material for: A possible space-based tsunami early warning system using observations of the tsunami ionospheric hole
Source: Sci Rep. 2016 Dec 1;6:37989. doi: 10.1038/srep37989 (PMC5131353; doi:10.1038/srep37989)
Supplement: Supplementary Information [file srep37989-s1.doc]

**Supplementary information**

**A possible space-based tsunami early warning system using observations of the tsunami ionospheric hole**

Masashi Kamogawa ^1^, Yoshiaki Orihara ^1^, Chiaki Tsurudome ^1^, Yuto Tomida ^1^, Tatsuya Kanaya ^1^, Daiki Ikeda ^1^, Aditya Riadi Gusman ^2^, Yoshihiro Kakinami ^3^, Jann-Yenq Liu ^4^, and Atsushi Toyoda ^5^

^1^ Department of Physics, Tokyo Gakugei University, Tokyo, Japan

^2^ Earthquake Research Institute, the University of Tokyo, Japan

^3^ Department of Engineering for Innovation, National Institute of Technology, Tomakomai College, Japan

^4^ Graduate Institute of Space Science, National Central University, Chung-li, Taiwan

^5^ Nuclear Safety Research and Development Center, Chubu Electric Power Co., Omaezaki, Japan

**a b**

**
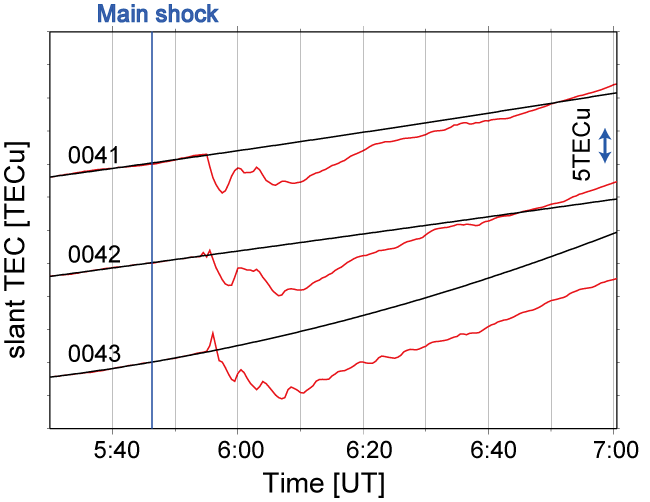

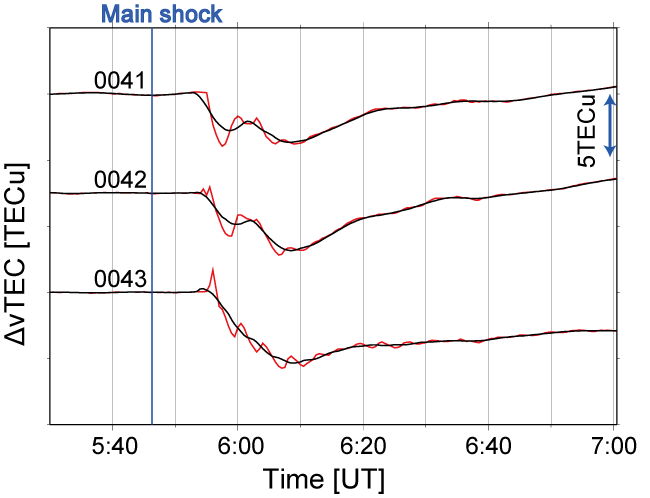
**

**Figure S1.** Data analysis to extract the TIH components. In this figure, the Tohoku EQ case is shown as an example. The vertical blue bars indicate the time of the main shock of the Tohoku EQ. Three receiving stations, 0041, 0042 and 0043, are illustrated. The GPS satellite for this data is #23. (a) Time-series of slant TEC (red) and fitting curve (black) obtained using the least-squares method. (b) Time-series of ΔvTEC and LPF ΔvTEC

**
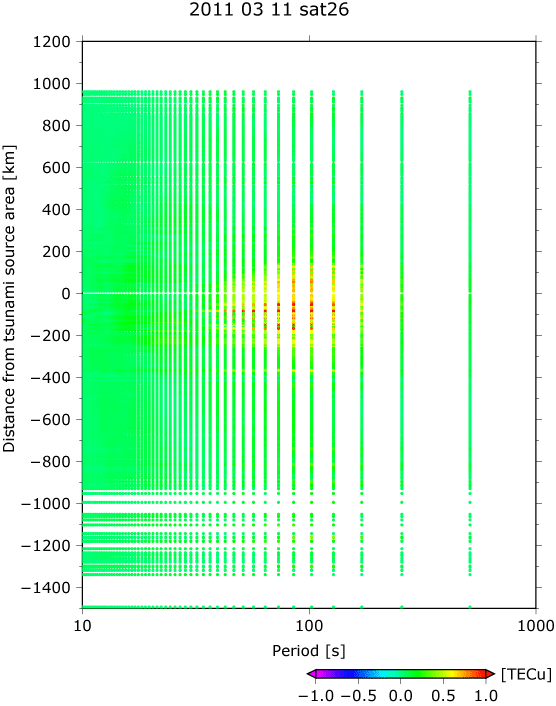
**

**Figure S2.** FFT analysis of ΔvTEC time-series for most of the receiving stations in Japan. The colour contours indicate the power spectrum. The vertical axis is the epicentral distance from the northern (positive) and southern (negative) receiving stations and the horizontal axis is the period. The GPS satellite for this data is #26. The period of time series was 1024 s which was ±512 s from the main shock.


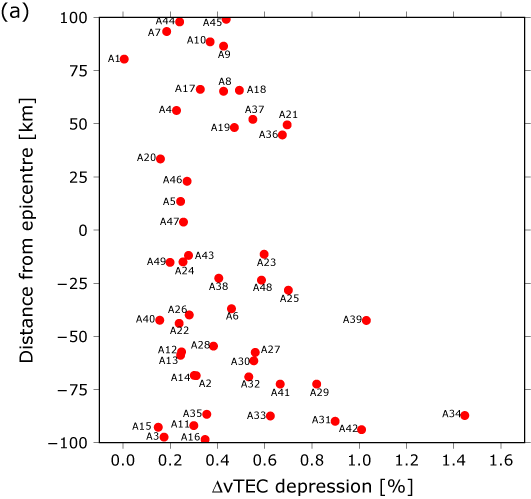

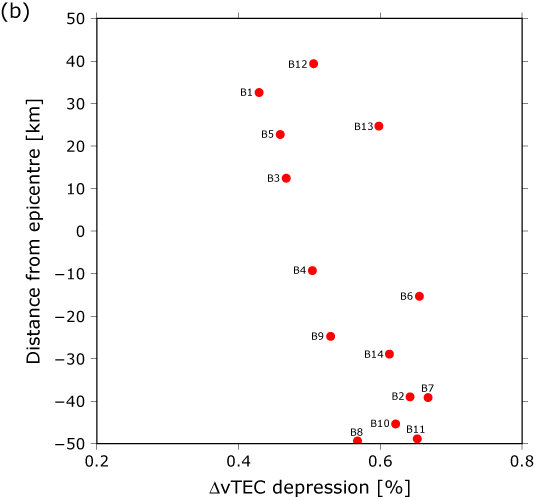

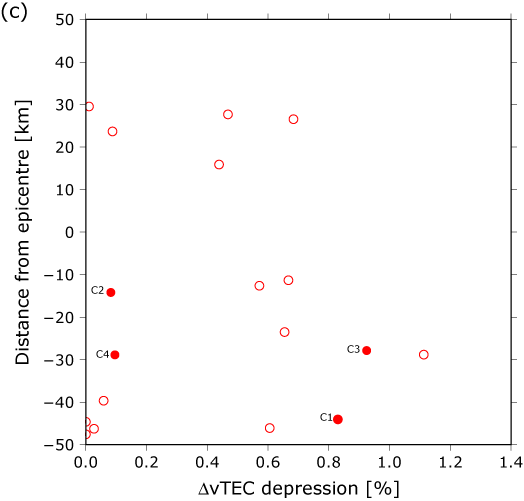

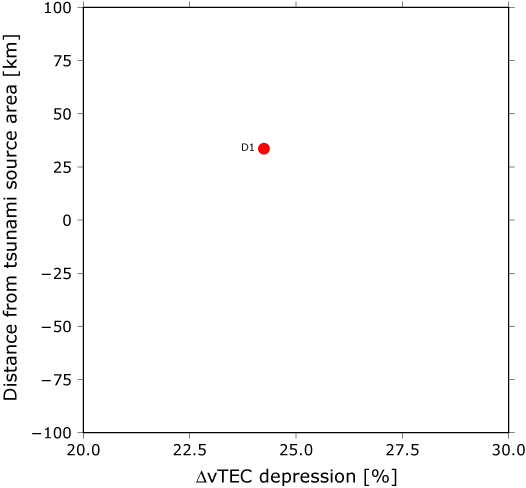


**
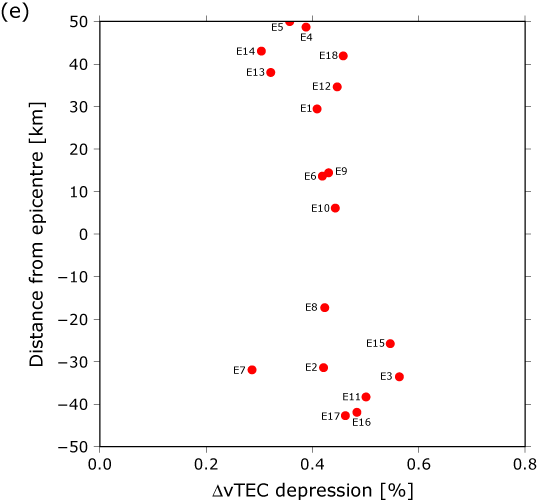

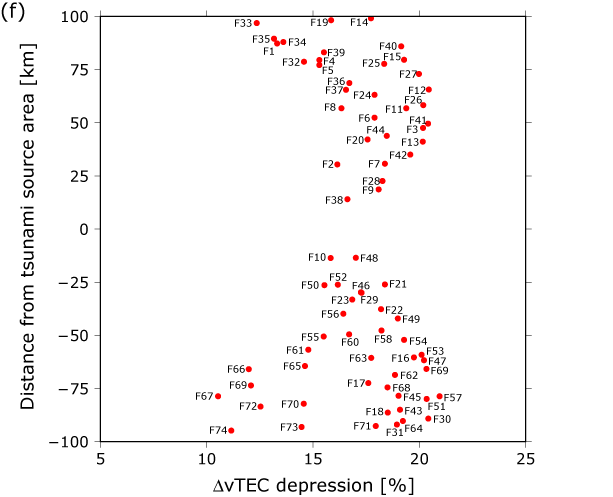

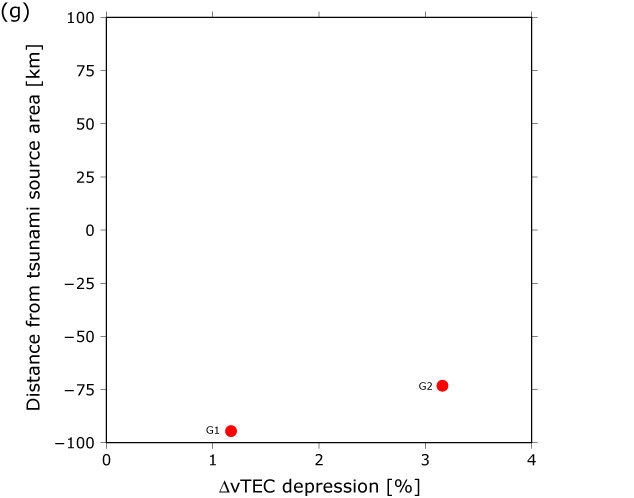
**

**Figure S3.** Percentage ΔvTEC depression versus epicentral distance from northern (positive) and southern (negative) SIP. Epicentral distance is shown 7 min after the mainshock. Solid red circles denote the percentage ΔvTEC depression inside analysed area. We note that some of data denoting open red circle in the Niigataken Chuetsu-oki EQ were removed for the analysis, because unrecognizable percentage ΔvTEC depression existed. Open Alphabet with the numbers corresponds to the time series shown in Figure Supplementary Fig. 4. (a) the Tokachi-Oki EQ, (b)the Off the Kii peninsula EQ, (c) the Niigataken Chuetsu-oki EQ, (d) the Maule EQ, (e) the Sanriku EQ, (f) the Tohoku EQ, and (g) the Illapel EQ.


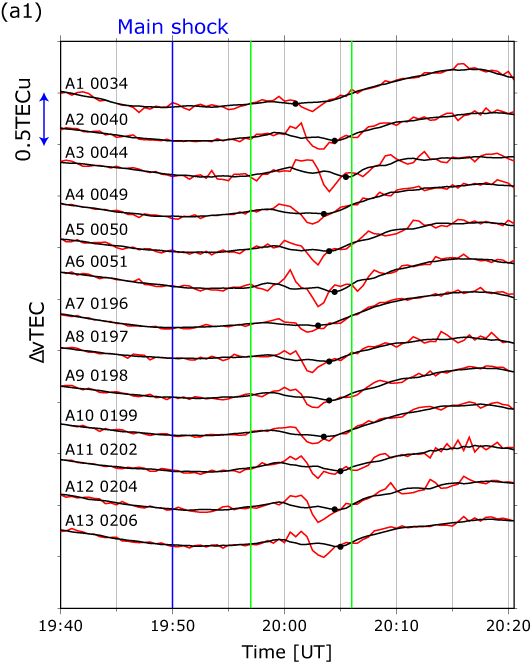

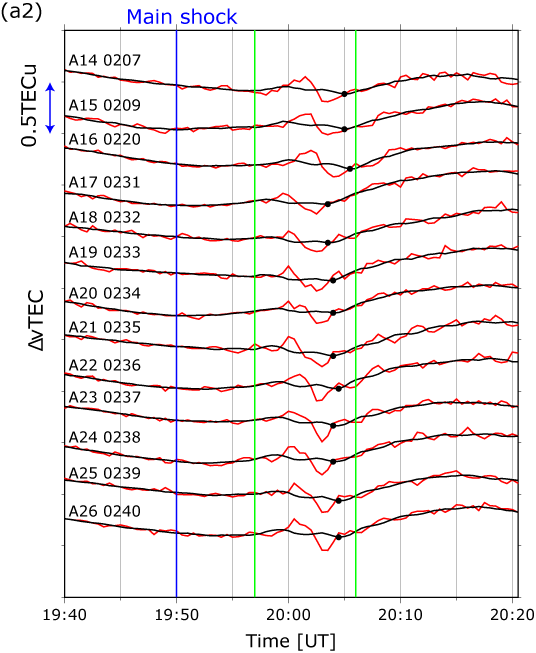

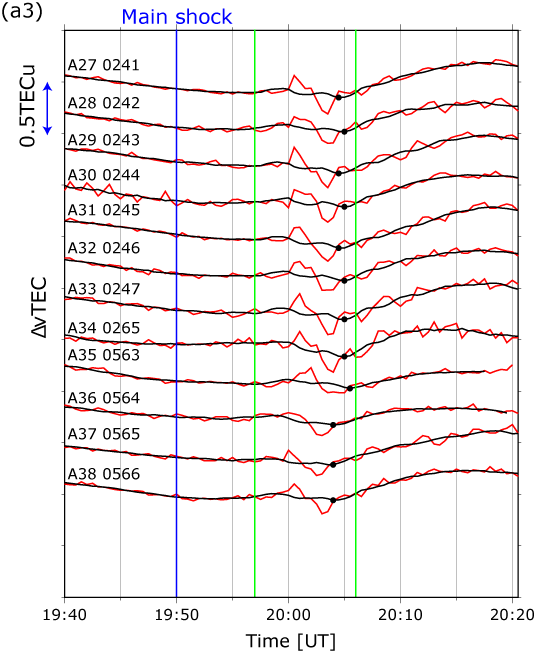

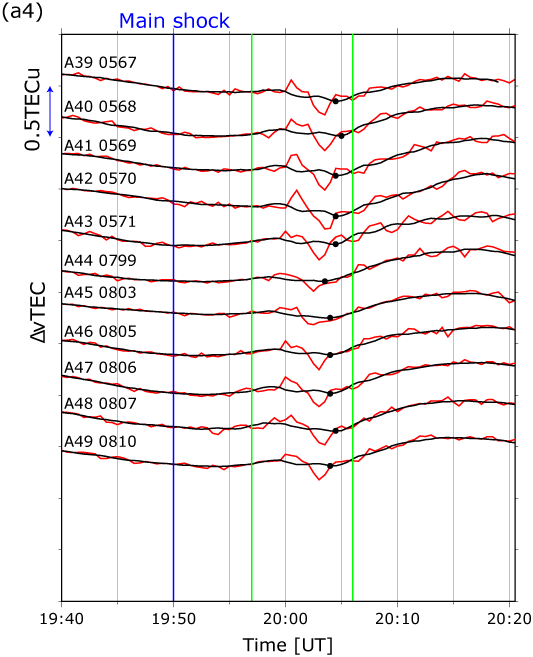

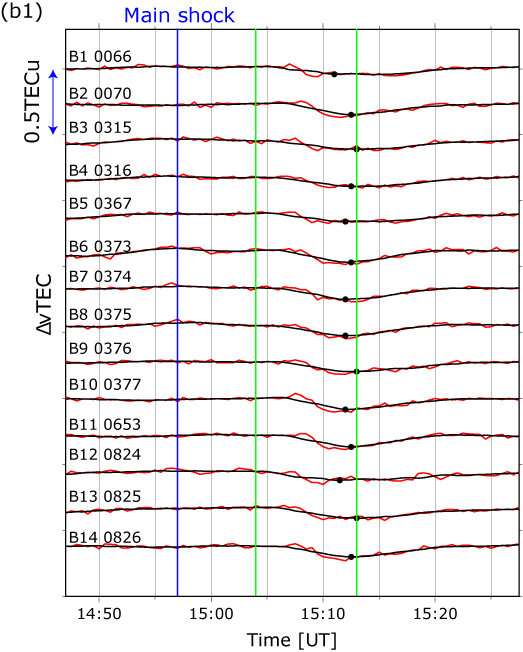

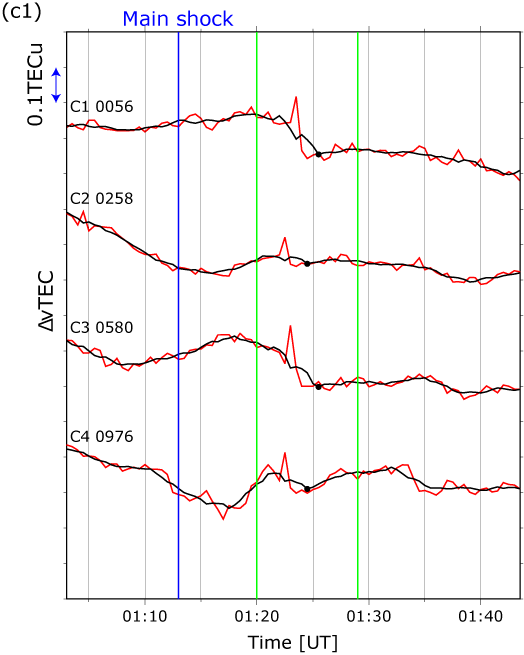


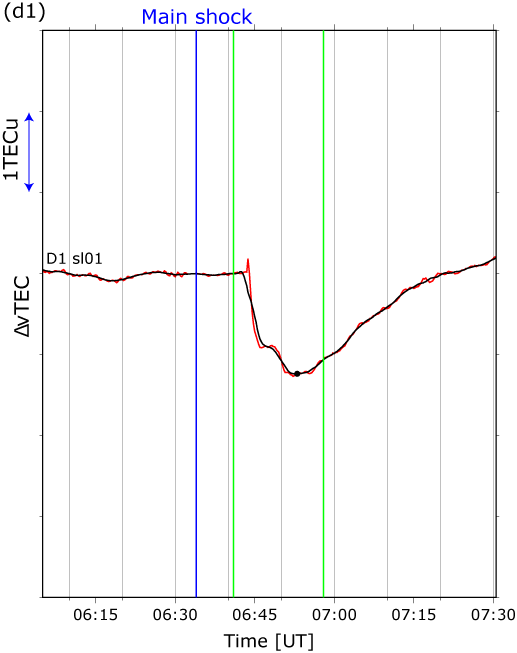

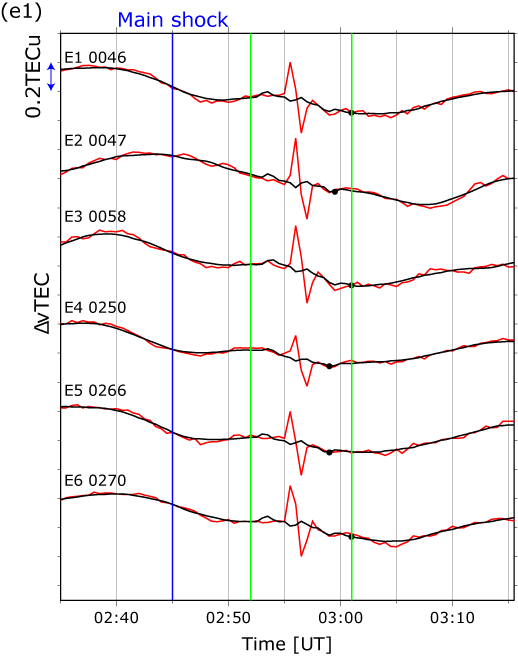

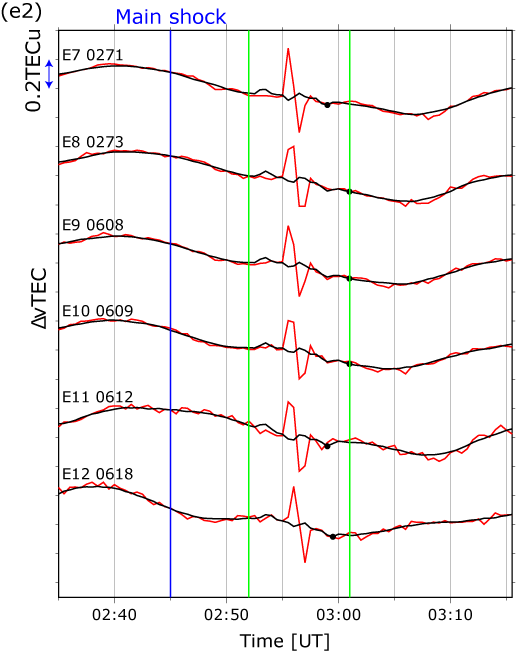

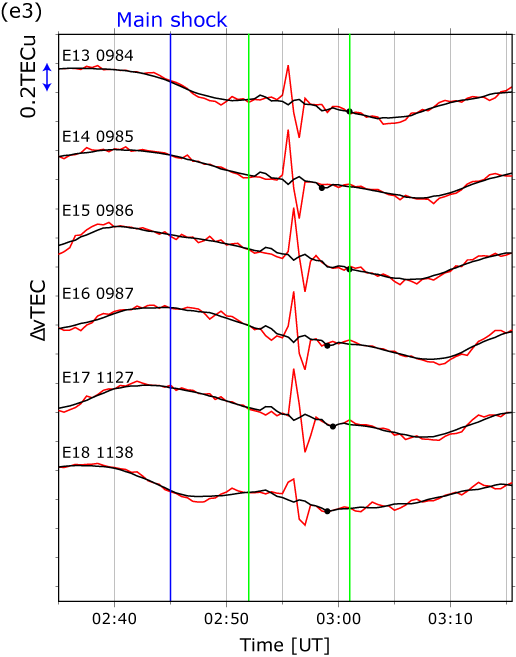


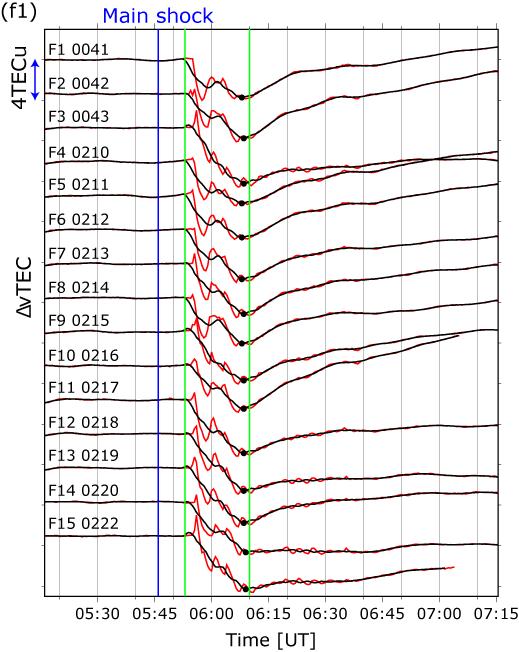

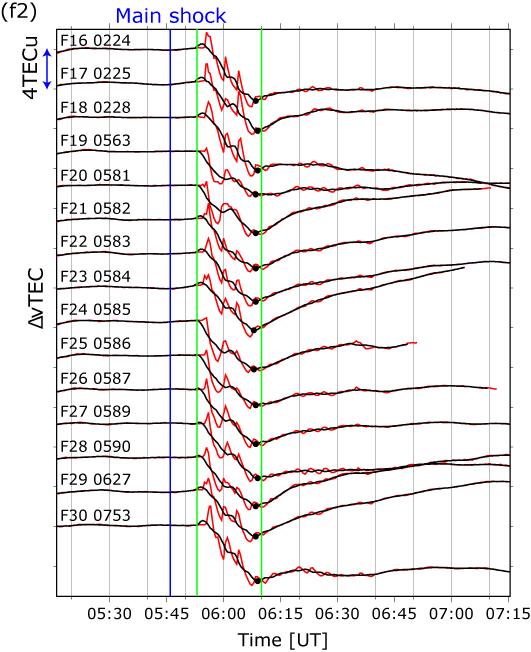

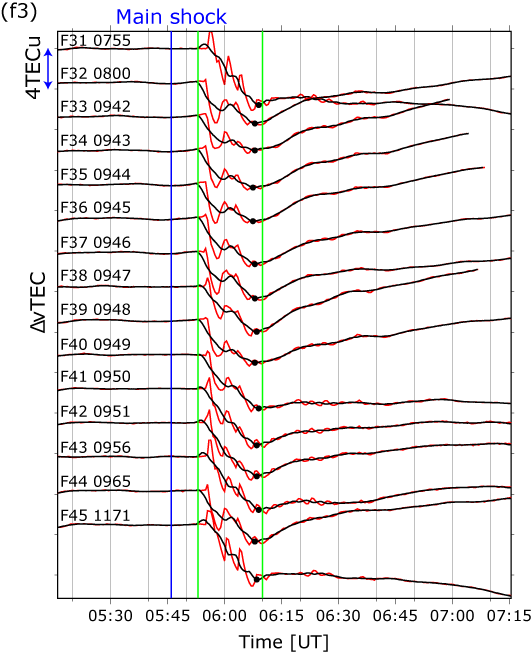

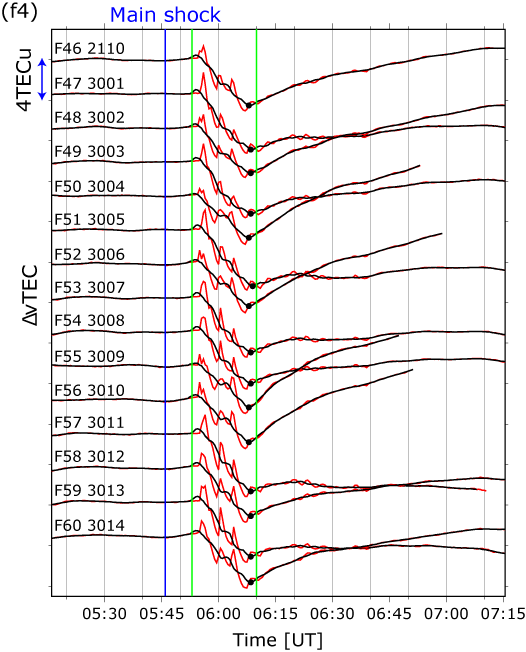

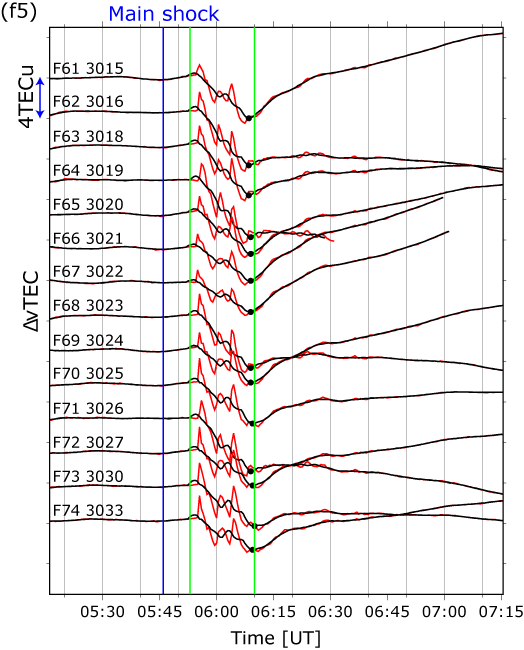

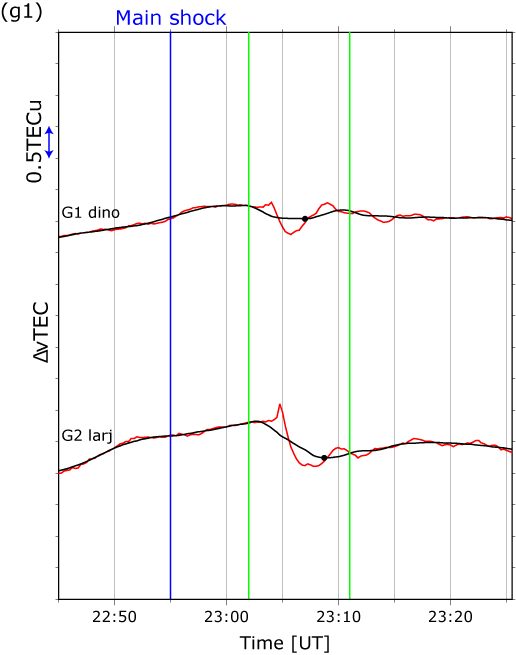


**Figure S4.** Time-series of LPF ΔVTEC. Alphabet with the numbers indicates data corresponding to the red dots of Supplementary Fig. 3. Solid dots denote the minimum points of ΔvTEC. Blue and green bars represent the times of the main shock, the start- and end-time points for δvTEC^TIH^ and δT_min_. estimations. (a) the Tokachi-Oki EQ, (b)the Off the Kii peninsula EQ, (c) the Niigataken Chuetsu-oki EQ, (d) the Maule EQ, (e) the Sanriku EQ, (f) the Tohoku EQ, and (g) the Illapel EQ.

**
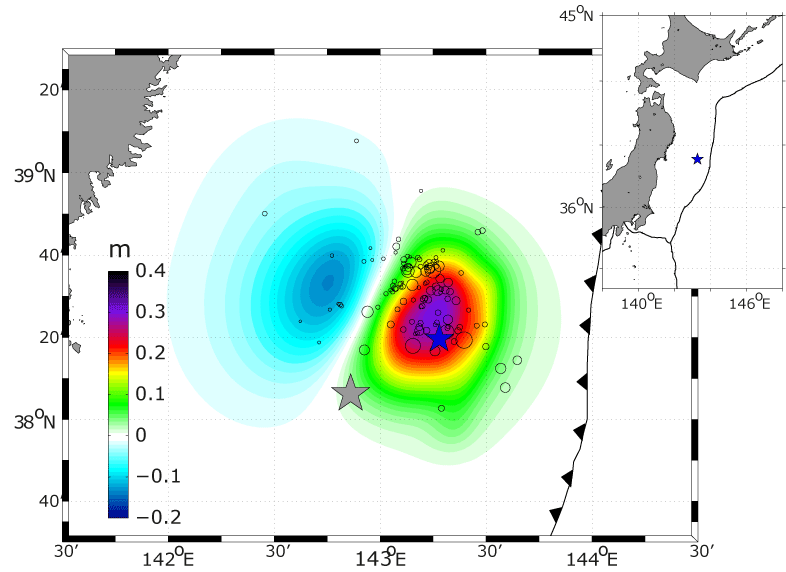
**

**Figure S5.** Initial tsunami height for the 9 March 2011 Off-Sanriku EQ (the largest foreshock of the Tohoku EQ). The blue star represents the epicentre of the largest foreshock, circles mark the epicentres of the foreshocks and the grey star indicates the epicentre of the main shock (the 11 March 2011 Tohoku EQ). The map was drawn using GMT 5 (http://gmt.soest.hawaii.edu/).
